# Human adaptation to biodiversity change: An adaptation process approach applied to a case study from southern India

**DOI:** 10.1007/s13280-019-01225-7

**Published:** 2019-09-13

**Authors:** Thomas F. Thornton, Rajindra K. Puri, Shonil Bhagwat, Patricia Howard

**Affiliations:** 1 School of Arts & Sciences, University of Alaska Southeast, Juneau, AK 99801 USA; 2 Environmental Change Institute, University of Oxford, School of Geography & the Environment, Oxford, OX1 3QY UK; 3 School of Environment and Conservation, University of Kent, Canterbury, UK; 4 Faculty of Arts & Social Sciences, The Open University, Milton Keynes, UK; 5 Wageningen University, Wageningen, The Netherlands; 6 University of Kent, Canterbury, UK; 7 School of Anthropology and Conservation, University of Kent, Marlowe Building, Canterbury, CT2 7NR UK; 8 Geography Discipline and Open Space Research Centre, The Open University, Walton Hall, Milton Keynes, MK7 6AA UK; 9 Department of Social Sciences, Wageningen University and Research Center, Hollandseweg 1, 6706 KN Wageningen, The Netherlands

**Keywords:** Adaptation, Biodiversity change, Climate change, Invasive plants, Vulnerability

## Abstract

Adaptation to environmental change, including biodiversity change, is both a new imperative in the face of global climate change and the oldest problem in human history. Humans have evolved a wide range of adaptation strategies in response to localised environmental changes, which have contributed strongly to both biological and cultural diversity. The evolving set of locally driven, ‘bottom-up’ responses to environmental change is collectively termed ‘autonomous adaptation,’ while its obverse, ‘planned adaptation,’ refers to ‘top-down’ (from without, e.g. State-driven) responses. After reviewing the dominant vulnerability, risk, and pathway approaches to adaptation, this paper applies an alternative framework for understanding human adaptation processes and responding more robustly to future adaptation needs. This *adaptation processes*-*to*-*pathways* framework is then deployed to consider human responses to biodiversity change caused by an aggressive ‘invasive’ plant, *Lantana camara* L., in several agri-forest communities of southern India. The results show that a variety of adaptation processes are developing to make Lantana less disruptive and more useable—from avoidance through mobility strategies to utilizing the plant for economic diversification. However, there is currently no clear synergy or policy support to connect them to a successful long-term adaptation pathway. These results are evaluated in relation to broader trends in adaptation analysis and governance to suggest ways of improving our understanding and support for human adaptation to biodiversity change at the household, community, and regional *livelisystem* levels, especially in societies highly dependent on local biodiversity for their livelihoods.

## Introduction

Human adaptation to environmental change is both a new imperative in the face of climate change and the oldest problem in our species’ history (Smithers and Smit 1997; NRC 1999; Janssen and Ostrom 2006; IPCC 2014). Human societies have always been subject to risks and vulnerabilities posed by changes in their material circumstances as a result of social, economic, ecological, and other environmental factors (Moran 2008). The diverse processes by which societies have dealt with social and environmental change throughout their history on the land and sea are well established in the scientific literature (Fagan 2008; Leichenko and Eisenhauer 2017). Humans have evolved a wide range of strategies in response to localised environmental changes, which have contributed strongly to specific social and ecological developments, including both biocultural diversification and homogenization (Smithers and Smits 1997; Moran 2008). The evolving set of locally driven, ‘bottom-up’ responses to environmental change is often collectively termed *autonomous adaptation* (Carter et al. 1994), while its obverse, *planned adaptation*, is typically used to reference ‘top-down’ (from without or State-driven) efforts to adjust a society, community or social-ecological system to existing or anticipated environmental change, as in climate adaptation (Fankhauser et al. 1999; Howard and Pecl unpublished results).

The term *autochthonous* may be preferable to autonomous adaptation, but has not yet been adopted into common use (Howard 2009). While autochthonous means indigenous or native, thus of local origin, autonomous means independent, without outside control, and/or self-governing. The latter thus neglects the interdependence between people and ecosystems on both spatial and temporal scales, and the lack of control that many otherwise autochthonous peoples have over drivers of change. This neglect of the autochthonous dimension of local adaptation and control is reflected in the IPCC’s current definition of autonomous adaptation, which is equated with ‘spontaneous adaptation’ and defined as, “Adaptation in response to experienced climate and its effects, without planning explicitly or consciously focused on addressing climate change” (IPCC 2014, p. 838). Such a definition denies all forms of conscious local or autochthonous movements, such as revitalization movements—“deliberate, organized, conscious effort by members of a society to construct a more satisfying culture” (Wallace 1956, p. 265)—which may be both autonomous and planned.

The current emphasis on top-down or planned adaptation denies both the capacity for organised responses that transform societies from within, as well as the inevitability and importance of local responses to change, whether bottom-up or top-down driven, where it is local responses that predominantly shape ecosystems and cultures and strongly influence socio-ecological systems at higher scales (Christoplos et al. 2009; Howard 2009).

After briefly conceptualising adaptation as an ongoing set of processes, and critiquing the dominant risk and vulnerability approaches to adaptation, we advance an alternative *adaptation processes*-*to*-*pathways* approach through the detailed analysis of responses to biodiversity change caused by an invasive plant, *Lantana camara* L. (‘Lantana’), in southern India. The paper draws on the conceptual framework developed for the Ecosystem Services and Poverty Alleviation project, Human Adaptation to Biodiversity Change, as described in Howard and Pecl (unpublished results). In so doing, we aimed to reconceptualise human adaptation in relation to surrounding ecosystem processes, *livelisystems* (sensu Dorward 2014), and human wellbeing. In our case study, we considered not only changing biodiversity itself, but also the relevant social, cultural, political and economic constraints and contextual factors that inform human responses to these environmental changes.

## Beyond risk and vulnerability: Adaptation as a set of interrelated and contingent processes

Human adaptation to environmental change is best understood over long temporal scales. The pace of environmental and social change is often slow and multigenerational, although it may become rapid when societal or planetary boundaries, or system thresholds (so-called *tipping points*), are exceeded (cf. Rockström et al. 2009; Raworth 2012, 2017; Howard 2013; Steffen et al. 2015). Similarly, localised plant and animal communities may take time to adjust to changes in climatic conditions. Over time, these shifts are manifested in changes in the structure, health, and diversity of ecological communities (Walther et al. 2002; Campbell et al. 2009). The critical nexus for human adaptation, then, is not so much change in global temperature or precipitation regimes, but rather the consequent and relevant local changes in biodiversity that support the web of life. As discussed in Howard (unpubl. results), species’ invasions can occur in a very short time frame, and can also provoke rapid human responses—thus providing a ‘real-time’ prospective for analysing human adaptation to biodiversity change.

Humans’ individual and collective capacities and actions to maintain wellbeing within a livelisystem depends on a particular diversity of agents, resources, and environmental processes. A livelisystem is defined as ‘a combination of the functions provided by assets (or resources) and activities undertaken in and by open, structured, and actively self-regulating systems’ (Dorward 2014). This concept expands and elaborates on the notion of *sustainable livelihoods* (Chambers and Conway 1992; Scoones 2015) within a social-ecological systems (SES) framework (Knutsson and Ostwald 2006). In doing so, the livelisystems concept allows for more robust incorporation of environmental change, resilience, and adaptation processes within socio-ecological systems (SES) analysis.

SES, including livelisystems, are inherently dynamic (Gunderson and Holling 2002; Berkes et al. 2003; Dorward 2014), thus human success requires strategies for resilience (Gunderson 2000; Folke 2006). This includes coping with temporary shocks, surprises, or other sources of stress and uncertainty, as well as more fundamental, long-term adaptations to systemic changes (Hollings 1973; Berkes et al. 2003). Coping does not always lead to adaptation and, in some cases, it may hinder adaptive pathways (Thornton and Manasfi 2010). In addition, adaptation processes are multiple, dynamic, and contingent, often making them hard to negotiate and implement, irrespective of whether they originate as planned or unplanned responses to climate change or other impacts.

Recognition of the dynamic and contingent nature of human adaptation to environmental change within SES has led many to advocate an *adaptive management* approach that is systemic, yet flexible, and capable of learning in response to feedbacks (Holling 1978; Berkes et al. 2000; MEA 2005). The future is not predictable. Uncertainty and risk abound, and change is inevitable, so adaptation cannot simply be planned ‘top down,’ but must continuously develop from the bottom and the meso-organizational levels of human societies as well. In other words, individuals and households must adapt, as must communities and states, to sustain themselves in a changing environment characterized by multilevel interactions and impacts of both environmental change and adaptation on SES (Howard unpubl. results). ‘Managing’ adaptation in a globalised world thus necessarily involves connecting these levels and their constituent actors, pathways, and institutional nodes.

In the environmental change literature to date, there have been three dominant approaches to the pursuit of adaptive management: one focused on social vulnerability (Smit and Wandel 2006), a second on risk management (Sarewitz et al. 2003) (both of which are based in disaster studies), and a third on adaptation pathways (Leach et al. 2010). The vulnerability approach involves assessing the status of key assets and capacities in a community or SES that are considered vital to its ability to cope or adapt, such as access to food, health services or credit, and strengthening those dimensions (Smit and Wandel 2006). One critique of this approach is that, while external vulnerability analyses might suggest adaptation pathways, these may overlook how people are actually adapting on the ground (Berrang-Ford et al. 2011). Another criticism is that this approach often reduces vulnerability to the status of technical or material asset ‘deficiency,’ rather than considering vulnerability as a possible systemic condition or process caused by an oppressive or extractive political economy (Cameron 2012). As Ribot (2014, p. 667) observes, this framing

of vulnerability and security as matters of access to assets and social protections…[overlooks their] context-contingent causal chains. A key recursive element in those causal chains is the ability – means and powers – of vulnerable people to influence the political economy that shapes their assets and social protections. Vulnerability is, as Sen rightly observed, linked to the lack of freedom – the freedom to influence the political economy that shapes these entitlements.

Such systemic political-economic disparities can present major barriers to adaptation and may constitute pathways of continued vulnerability (Pahl-Wostl 2009; Pelling 2011; Marino and Ribot 2012; O’Brien 2012).

The risk management approach to climate adaptation seeks to reduce exposure to environmental change by applying formal assessment methods and tools that can identify probable threats to human wellbeing. Adaptive response to these threats, which may include events such as floods, droughts and heatwaves, is achieved through optimization of risk-reduction benefits versus costs (World Bank 2010), within the bounds of probabilistic uncertainty (Borgomeo et al. 2014) and acceptable risk at the societal level (Oels 2013). As is the case with some vulnerability approaches, risk analyses may go beyond ex-ante methods to include participatory and social instruments to determine perceptions of and preferred responses to risk in local populations (Van Aalst et al. 2008). Sarewitz et al. (2003, p. 810) conclude that both risk and vulnerability approaches are necessary for effective planning, since ‘a myopic focus on risk to the exclusion of vulnerability can easily enhance rather than reduce the prospects for negative outcomes.’

However, some scholars take issue with the tendency of both vulnerability and risk analyses to swiftly hone in on an inappropriately narrow set of alternatives configured for the purposes of making uncertainty ‘manageable’ within cost–benefit and risk equations. Instead, policy processes should be more equitable and open to a wider diversity of participants, ideas, and values in decision-making (cf. Stirling 2006; Leach et al. 2010; O’Brien and Wolf 2010; Van Ruijven et al. 2014). Only by widening adaptation planning beyond existing managerial and sociotechnical paradigms, as well as beyond climate change itself (Forsyth and Evans 2013), can more transformative pathways to change and sustainability be realised.

Such critiques have led to a third major orientation to adaptation termed the *pathways approach*. It begins by recognising that existing governance structures are often ill-equipped (due to lock-in, vested interests, capacity, etc.) to imagine or deal with the need for alternative, transformative trajectories, thereby constraining adaptation actions to existing pathways of response to environmental stressors. Existing pathways are likely to be insufficient or inefficient in the face of unprecedented impacts wrought by environmental change, or may reinforce unsustainable and inequitable development processes. Sustainable solutions must often address multiple issues on multiple levels (or pathways) at the same time, as argued, for example, in the case of *climate*-*smart agriculture* (CSA), which supports innovations that simultaneously adapt for climate change, reduce GHG emissions and insure food security (Taylor 2017). Adaptive pathways to sustainability are invariably negotiated within dynamic, complex systems of social, environmental, and technological processes, which likely require multiple diverse innovations in human cultural, social, and material realms in order to effect transitions to sustainability (Smith et al. 2005; Geels and Schot 2007; Leach et al. 2010). Adaptation pathways are thus contingent on ‘alternative possible trajectories for knowledge, intervention and change, which prioritise different goals, values and functions’ in decision-making (Leach et al. 2010, p. 5). ‘Pathways thinking,’ its proponents argue, allows adaptation to be reconceptualised so that actions on climate change can be linked with transformative social change at multiple levels (Wise et al. 2014: 327). However, the pathways approach has yet to be consistently applied or assessed in diverse case studies; nor has it been linked to broader human adaptation processes, as we propose here by wedding it to an existing scheme first proposed by Thornton and Manasfi (2010).

### Developing an autochthonous adaptation process approach

Yet another approach, elaborated upon here, is to focus on actual autochthonous, rather than planned, adaptation processes and their various local articulations in relation to specific social and environmental constraints and changes over time. This *adaptation processes approach* avoids some of the limitations of the vulnerability and risk perspectives, which too often remain narrowly focused on extreme environmental events and short-term institutional responses, or coping strategies, and thus overlook important ongoing autochthonous adaptations (Thornton and Manasfi 2010). Moreover, our approach complements the pathways approach by identifying eight major ongoing human adaptive processes that enable incremental and transformative adaptation pathways: (1) mobility, (2) exchange, (3) rationing, (4) pooling, (5) diversification, (6) intensification, (7) innovation, and (8) revitalization (Thornton and Manasfi 2010). The utility of this approach is that it provides a basic, universal analytical framework for assessing a full range of human adaptation pathways in response to environmental change, without reifying any single adaptation driver, scale, sector, process, pathway, or institution. There is also ample space to develop relevant subcategories (e.g. Howard unpubl. results). For example, mobility could be divided into temporary or permanent migration versus resource tracking, exchange could be further categorised into generalised versus balanced or negative reciprocity, diversification into substitution, hybridization, and so forth. Moreover, the adaptation processes approach is compatible with decentralised and participatory orientations to adaptation governance and capacity building. It provides a comparative means of assessing both the appropriateness of adaptation policies and the continuing viability of local knowledge, values, and practices that have contributed to the resilience and adaptive capacity of communities over longer time scales. The adaptation process framework is now being constructively applied in a variety of geographic, livelihoods, and governance contexts in Asia, Africa, and the Americas (e.g., McDowell et al. 2014; Thorn et al. 2015; Thornton and Comberti 2017).

The adaptation processes approach also addresses a neglected issue in present adaptation studies: how existing top-down governance, or ‘planned’ adaptation measures, may effectively limit or subvert local autochthonous adaptation through policies of encroachment, appropriation, development, and conservation (e.g. Nair 2014; Macura et al. 2016; McKinnon et al. 2016; Howard unpubl. results). Indigenous peoples, ethnic minorities, and subsistence populations often have little choice but to ‘adapt’ to the dominant socio-political system and its objectives for growth and ‘progress.’ At present, these uncomfortable political and historical-ecological determinants of social-ecological systems tend to be elided in favour of more depoliticized (Ferguson 1994) and often spurious (Thornton and Manasfi 2010) top-down sociotechnical, or so-called ‘neoliberal’ market-based approaches to managing adaptation and development. Even where, following Olsson’s (2006) model of transition to adaptive governance, it is recognised that (1) the existing management system is in crisis; and (2) a ‘transition’ to a new regime is necessary, the crucial third phase of discovering, changing, and ‘institutionalizing’ new regimes presents a huge challenge, in part because autochthonous adaptation processes either go unrecognized or are actively undermined as a consequence of colonialism, development, state-formation, and globalization (Leichenko and O’Brien 2008). Yet, from our perspective, genuine (as opposed to spurious) adaptation means considering the full range of human adaptation responses at hand, where the disproportionate impacts and burdens placed on local peoples may be alleviated or reversed by expanding their opportunities to pursue a fuller range of adaptation processes and pathways.

In this respect, the adaptation processes approach is compatible with the pathways approach outlined above, while at the same time expanding the repertoire of adaptation processes that can contribute to, redirect, or transcend narrow pathways. Thus, in the case study that follows, we combine the two into an integrated *adaptation processes*-*to*-*pathways* approach.

### Applying the adaptation process framework in a case study in South India

We applied the *adaptation processes*-*to*-*pathways* framework to a case of rapid human adaptation to biodiversity change, specifically, to the proliferation of the invasive plant *Lantana camara*. (‘red sage’ or ‘lantana’) in Karnataka, southern India. We chose this case of established but ongoing biodiversity change because Lantana’s impacts are prolific and profound, and thus is reflective of the kind of environmental changes to which humans have long been contributing and adapting (Bhagwat et al. 2012; Howard 2013).

**Fig. 1:**
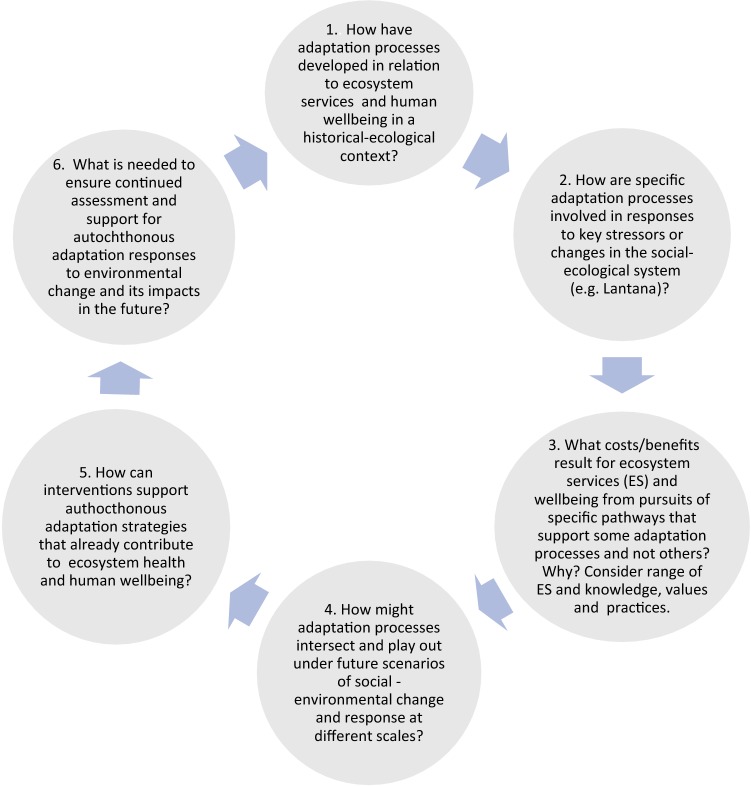
Adaptation development cycle mindful of autochthonous processes

We developed a working conceptual framework for the research (Howard 2009) that focused data collection on local knowledge of Lantana and its impacts, value changes in key natural resources and practices as a result of Lantana impacts, and livelisystem responses as key aspects of adaptation processes. However, biodiversity is only one of a range of risks and stresses that people must manage, such as poverty, poor health, depressed commodity markets, reduced access to land and natural resources, and increased water scarcity. Similarly, adaptation processes are multiple and intersecting, and involve not only explicit and tacit strategies for managing change in a particular species such as Lantana, but also the wider knowledge, values, practices, and institutions deployed when managing risk, stress, and uncertainty at household, landscape, and SES scales (Orlove 2005; Howard 2009; Clarke et al. 2013; Dutra et al. 2018; Howard unpubl. results). These complexities tend to be ignored or oversimplified in conventional risk and vulnerability analyses and decision chains. In contrast, our case study explores these complexities in detail, leading to an alternative, more dynamic and contingent rendering of the adaptation cycle, based on an *adaptation processes* model of response to environmental change (Fig. 1).

### Study area and methods

The spread of Lantana has been well documented in the Western Ghats region of India since the late nineteenth century (cf. Bhagwat et al. 2012; Kannan et al. 2013). Lantana’s ‘invasion’ of the Male Mahadeshwara Hills (MM Hills) Wildlife Sanctuary, southern Karnataka, is illustrative of the kind of rapid biodiversity change that is already occurring across India and will accelerate with climate change, causing significant declines of key species and shifts in local biodiversity and ecosystem dynamics. Failure to adapt to Lantana’s rapid spread could lead to long-term stress or even collapse of key parts of the current SES.

Employing a range of methods, we collected data on perceptions and responses to Lantana by residents and officials stationed in the MM Hills region. We used historical-ecological records combined with field and household surveys, participant observation, and semi-structured and casual interviews to begin to understand the trajectory of the Lantana invasion, its effects, and how people respond and adapt to its impacts on their forest and field ecosystems. The MM Hills region was chosen in part because it is an ecologically diverse, multi-ethnic region in which people engage in a wide range of livelihood activities, including hunting and gathering, cattle and goat herding, small-scale agriculture, and wage labour, nearly all of which are affected by Lantana’s spread. Correlatively, local households and communities are evolving a broad range of strategies to adapt to this biodiversity change. We were fortunate to work with Ashoka Trust for Research in Ecology and the Environment (ATREE) scientists, especially the late Dr. Ramesh Kannan, already engaged in long-term research in the area on the ecological impacts and human responses to Lantana. ATREE has developed an award-winning programme to use Lantana in a small-scale furniture enterprises (Kannan et al. 2009). In conjunction with ATREE, we sought to build upon their research on local adaptation strategies (e.g. Shaanker et al. 2010; Kent and Dorward 2012; Kannan et al. 2014; Puri 2015; Kannan et al. 2016).

Our study was centred in the village of Kombudikki and surrounding communities in the MM Hills, where the majority population is Lingayat. Lingayat residing in this region are vegetarian cultivators dependent on finger millet (*Eleusine coracana* (L.) Gaertn.) as their primary staple. Many also own and graze cattle and goats, and forage for non-timber forest products (NTFPs) for consumption and exchange, as well as engaging in wage labour. Soliga are a distinct tribal minority in the area (a ‘Scheduled tribe’ in Indian government classification), typically occupying more marginal lands in smaller household units with a higher degree of direct dependence on forest resources (such as bamboo) for their livelihoods (Si 2016). In addition to cultivating millet and beans, grazing goats and cattle, and collecting honey and plants, Soliga are also employed in part-time wage labour in nearby quarries and other industries. The landscapes, livelihoods, and dwellings of the two groups are very much intertwined and, not surprisingly, their views on Lantana’s spread are similar (Kent and Dorward 2015).

Households from both groups who are below the poverty line receive subsistence support through India’s Public Distribution System. In 2013, the MM Hills area was declared a Wildlife Sanctuary, and there is a concerted effort by the Forestry Department to more strictly regulate access to forests, including sales of NTFPs and animal grazing. Cowsheds, or forest corrals, have been forbidden, thus only daily grazing of cattle and goats is permitted in the forests.

## Local knowledge of Lantana, its spread and its impact

Both groups view Lantana as a recently aggressive invasive, consistent with the literature (Shaanker et al. 2010). Adults can remember a time when Lantana was ‘not so bad’ or prevalent only in certain dry, deciduous regions on the forest edge, but now it is said by many to be ‘everywhere,’ meaning in virtually every ecozone they inhabit. More nuanced assessments were given by cattle herders, who were aware of places where the plant’s spread had slowed, stopped, or even been reversed, but these were typically small microhabitats within the forest supporting deep canopy cover, or where Lantana competes with plants such as *Cassia fistula*, another invasive, in and around fields and other cultivated areas.

The local and traditional knowledge that we documented was largely consistent with the existing literature on the negative effects of Lantana-related biodiversity change on ecosystems (Table 1).

**Table 1:** Impacts of Lantana on Ecosystem Services (adapted from Shaanker et al. 2010) (* also identified by locals we interviewed; ** added by our local facilitators)

Positive effects	Negative effects	Anomalies
Increase in regeneration of native species Increase in soil nutrient pools and nutrient mobility Increase in soil nutrients (when burned in fields)* Increase in regeneration of non-timber forest products (NTFPs) Antifungal potential in soil Antimicrobial, fungicidal, insecticidal, and nematicidal activity, but not antiviral activity Lantana pulp is used for writing and printing paper Used as a cover crop in deforested areas and also to enrich the soil and protect against erosion Fruits provide food for frugivorous birds, barking deer, sambar deer, sloth bear**	Decrease in regeneration of native species* Decrease in biodiversity* Decrease in species richness* Contamination of native Lantana species gene pool Threat to native Lantana species from competition* Reduces the pollinator loads of native plants Alters fire regime* Affects human health by harbouring malarial mosquitoes and tsetse flies Affects human health by harbouring ticks and mosquitoes** Allelopathic effects, resulting in either no growth or reduced growth* Decrease in community biomass and proportionate increase in foliage component in vegetation* Loss of pasture land* Threat to agriculture (high cost of clearing from fallow fields; threatens fallow system)* Poisoning of cattle, buffalo, sheep, goats, horses, dogs, cattle* Unripe fruit mildly poisonous* Consumes water, stressing local supplies and undermining local plant/animal communities** Standing water made non-potable by Lantana leaf fall** Flowers negatively affect taste of honey, an important NTFP** Access to forest and beneficial products and services is impeded**	Soapnut remains abundant in some areas invaded by Lantana* *Kole hullu* (fodder grass species: *Brachiaria setigera*) thrives under Lantana and is eaten by cattle** Burning of weeded Lantana in fallow fields is said to enhance fertility of soils** *Cassia fistula* seems to suppress or check Lantana growth** Large bamboo (*Bambusa bambos*) suppresses or checks Lantana growth**

Despite being labeled locally as *jedi gida* (‘useless plant’), local residents identified several positive ecosystem values associated with Lantana. With its abundant fruits, it contributes to feeding birds and other forest animals, such as sloth bears. Farmers enhance the fertility of their soils by burning weeded Lantana and applying the ash to fields before planting. It is also recognised for its beautiful and aromatic flowers, its value (both alive and dead) as excellent fencing material, and its potential use in manufacture. These positive ecosystem values suggest, from an adaptation perspective, that invasive species such as Lantana may be recognised early on for selected positive impacts on livelihoods, yet still not be highly valued overall (Howard unpubl. results). At present, Lantana is just beginning to be used to manufacture furniture and other products, so the realization of livelihood benefits is quite limited in comparison with the plant’s detrimental effects on other livelihood resources. At the same time, certain species are resistant or resilient to Lantana, such as soap nut (*Sapindus trifoliatus* L.), large bamboo (*Bambusa bambos* L.) and some grass species (e.g., *Urochloa*), which is significant for livelisystems that must adapt to reduced biodiversity and vegetative cover that is increasingly dominated by Lantana.

The negative effects of Lantana on biodiversity and ecosystem processes affect the value of key assets within the livelisystem. For example, soap nut’s value in the subsistence portfolio, as a commercialised NTFP, may increase as a result of its ability to maintain its abundance for harvesters despite Lantana’s invasion. On the other hand, commercialised NTFPs such as bamboo (specifically *Dendrocalamus strictus* L. which is woven into baskets, mostly for sale) and broomstick (*Phoenix sylvestris* Roxb. which is sold for broom manufacture) may be devalued as resources as they become too costly to collect or transform into products (e.g., bamboo baskets for sale) as Lantana reduces their abundance, quality, or ease of harvest. Similarly, the labour required to tend cattle increases with the decline and fragmentation of fodder resources and reduced access to the forest due to Lantana’s thick spread, making it costly for households to maintain significant numbers of cattle as assets, despite the high prestige and practical value associated with these animals (Harris 1966).

It is important to acknowledge that these different positive and negative effects do not represent simple trade-offs. Livelihood strategies evolve over time and may be deeply embedded within a cultural system of values and assets that does not change as readily as biodiversity itself. Indeed, strong positive values towards a particular asset or livelihood strategy may mean that it will be pursued well beyond a level that is optimal or even sustainable from the standpoint of ecosystem services. At the same time, retaining a diverse portfolio of traditional livelihood assets and strategies, even at reduced levels, can enhance the transmission of traditional knowledge and values, while also strongly contributing to household and community resilience. Thus, bamboo and broomstick collecting and forest cattle grazing do not cease altogether with increased costs and shifting livelisystems. Rather, these resources remain as affordances (sensu Gibson 1979) and cultural assets in the forest ecosystem, and may be increasingly pursued by specialists, by those with fewer viable alternative livelihood strategies, or simply more opportunistically pursued as part of a general rebalancing of livelihood options (Kent and Dorward 2015). Such changes in socioeconomic orientations toward basic assets may themselves become adaptation strategies insofar as they maintain a functional continuity and diversity of cultural values and roles, or niches (Barth 1956), within a community in which individual opportunities, identities, and ‘careers’ (Goldschmidt 1990) may be shifting dramatically.

## Adaptation processes: Responses to Lantana and its impacts

Given our findings concerning the knowledge of invasive Lantana and its effects, positive and negative, on valued resources and livelisystem assets, we can now analyse responses to this biodiversity change using the *Adaptation Processes* approach in terms of the eight general processes, or modes, identified above. Our analyses are summarised in Table 2. We identify specific autochthonous strategies (developing or established) for each of the eight modal categories, from mobility to revitalization.

**Table 2:** Adaptation processes in relation to biodiversity changes associated with Lantana

Adaptation pathway	Status prior to Lantana invasion	Lantana effects	Livelisystem impacts and contingent adaptation effects on households
**Mobility** Seasonal movement to avoid risk or in search of better circumstances	Less time spent resource tracking in the forest with resource patches easier to find, less effort in grazing cattle, collecting NTFPs; May have been more competition for forest resources from greater number of HHs	Reduced forest access; Vulnerability to wildlife encounters/conflicts; Further to go to find resource patches and/or avoid Lantana patches until the number of HHs engaged in the practice decreases, due to moving cattle away or selling off herds	Reduced forest production (e.g. bamboo harvest); Reduced livestock and fodder; Increased sedentism/migration for other work, reliance on agriculture, public distribution system; Increased mobility/access costs to find suitable grazing fodder, increased danger of accidents
**Exchange** Flow of material and symbolic goods and services between people	Household less reliant on exchange, more reliant on diverse forest and forest-edge activities, including herding	Decreased health of livestock due to lack of quality fodder and poisoning from Lantana, according to some herders	Reduced exchange of forest products; Increased exchange of Lantana products; Pasture for livestock services (traction or manure) or for pasturing cattle on fallow fields; Selling off of livestock
**Rationing** Controlling circulation or consumption of limited or critical resources	Generalised grazing and harvest rights to forest resources; Household plots sufficient for agricultural production	Reduced grazing fodder; Reduced NTFP patches; Encroachment on agricultural fields	Changes in land and resource use patterns to avoid overgrazing & competition; Forestry Dept. restrictions on NTFPs; More cash crops; Fragmentation and degradation of agricultural land base
**Pooling** Sharing or linking of assets (wealth, labour, knowledge)	Less pooling of cattle for grazing, and more family-based herding	Increased costs associated with large herds and forest grazing	Pooling of herds and labour for grazing (including outsourcing care to herders elsewhere); Pooling of livestock and labour assets for agriculture services to cope with absent wage labour force
**Diversification** Increasing variety of assets and strategies to enhance livelihoods	Mixed economy based on agriculture (millet), livestock, and NTFP production, perhaps some wage labour	Lantana spread reduces other plants, alters habitats and ecosystem services; reduces opportunities for substitution	Shift into Lantana production for fencing, fertilizer (burned Lantana residue) and food (some eat berries); Some use for furniture and fuel wood. Increased reliance on wage labour & public distribution; Some loss of medicinal and wild herb use
**Intensification** Increasing the availability of resources by boosting yield within a certain space or time	Relative balance between agriculture, herding, and foraging	Lantana encroachment leads to shrinking & fragmentation of grazing, gathering, agriculture areas	Intensive weeding and eradication of Lantana around crops (millet) and paths; Increased reliance on microhabitats with low levels of Lantana for NTFP, grazing; Increased forest herding specialisation (among those not diversifying)
**Innovation** New, unplanned method or technique that arises to address a certain need	*Lantana indica* known as medicinal; foreign *Lantana camara* not used except as ornamental	Lantana has value for ecosystem and cultural services	Lantana furniture & basket production (NGO support); use for fencing, fertiliser and fuel; other product potential; Mitigation techniques for reducing conflicts with agriculture; Some new livestock regimes and herding patterns; Outsourcing of herding
**Revitalization** Organized reconfiguration of ideology and practice to reduce stress and create a more satisfying culture	No ideology or practices surrounding plant other than as ornament or weed	Realization that Lantana cannot be eradicated; adaptive management is necessary conceptualized as devil or foreign invader, perhaps rationalising impacts and locating blame	Reconfiguration of livelihoods towards adaptive avoidance and strategic use & valorisation of Lantana; Some revitalisation of inter-village ties for services and exchange to adapt to Lantana impacts on mobility, plant productivity and grazing

Across the region, many of these modes of adaptation were deployed specifically, if not exclusively, to adapt to Lantana. However, some adaptations were more productive than others. Innovation and valorization of Lantana, for example, has been slow to occur despite the top-down dissemination of knowledge and skills concerning its value as a material for furniture-making and other manufacture. To date, relatively few families have become involved in the evolving Lantana furniture industry initiated through an NGO intervention to support Soliga livelihoods (ATREE 2012), although some do collect it for sale as a raw material. This may be partly due to the fact that Lantana collection entails high labour costs and risks, including increased human–wildlife conflict associated with more limited forest access and mobility, but also due to distances to markets or furniture-making stations. While the Lingayat are currently excluded from participation in this social enterprise project, they appear to have no interest in establishing competing enterprises that use Lantana.

In most MM Hills communities, Lantana continues to be conceptualised as a relatively low value asset, although people do engage in government-paid labour to remove Lantana from road and forest trail edges. A strong positive or even neutral value orientation towards the invasive plant has yet to evolve in most segments of the population, including among those who are actively exploiting it as part of their livelihood portfolios. Anewala, among the most productive Lantana manufacturing villages in the MM Hills, may present an exception—yet, even in this community, the uptake of small-scale production of Lantana furniture and other goods appears to be limited to a few industrious families, and the community’s orientation toward the plant could hardly be characterized as positive. In addition to people’s own negative experiences with Lantana’s effects on their livelihoods and landscapes, NGOs’ negative attitudes toward ‘invasives’ may also contribute to locals’ adverse orientation towards the plant. This demonstrates that adaptation processes and pathways are not always synergized, and thus must be examined, as our model suggests, in their full and diverse local cultural and ecological contexts.

### Synergies and feedbacks of adaptation pathways

The pathways humans follow in adapting to biodiversity change lead to new contingencies and constraints on human–environmental interactions. Ongoing adaptations themselves affect biodiversity, ecosystem processes and human wellbeing (Howard unpubl. results). Results of the Lantana case show that adaptation to biodiversity change and in related ecosystem processes due to Lantana’s spread are having a major impact on people’s livelihoods and wellbeing. In terms of biodiversity, Lantana’s suppression of native plant species through successful competition for light, soil, pollinators, and water raises costs and limits peoples’ ability to use and manage native species as assets. Similarly, Lantana’s shrubby proliferation reduces general access to the forest, thus further limiting or, spatially and temporally, concentrating its use for livelihoods. Circumscribed use of a changing forest, in turn, may reduce the detailed, continuously updated landscape and ecosystem knowledge-building that comes with more regular engagement with the forest. Ultimately (and evidence for this is already emerging), for some segments of the population, alienation from the forest may lead to a gradual reconceptualization of forest environment itself: in the case of Lantana, from a landscape of productivity to one of contamination (of productive resources by foreign invaders or spiritual forces) and fear (of unseen dangerous wildlife, etc.). Such negative orientations make it less likely that Lantana will be positively valued as an economic resource unless a revitalization or innovation process, such as through a wider uptake of basket and furniture making, valorises the plant as a livelihood asset. In these and other ways, adaptation processes feedback on biodiversity changes.

Devaluation of the forest as a source of livelihood assets for forest peoples can bring about a crisis in knowledge, skills, identity, and occupations. Traditional forest knowledge and practices are no longer sufficient to guarantee wellbeing. New knowledge and skills involving wage labour in new environments, such as quarries, must be learned and valued. But does short-term labour in quarries or work in industries provide positive identity and career values? It is perhaps too early to tell. Nevertheless, it seems that the preferred mode of adaptation, particularly among males, is to diversify their livelihood pursuits so as to remain present at family agricultural holdings during key times of year, such as crop planting and harvesting, while taking up wage labour at complementary times of year when the agricultural workload is low. This diversification, as well as the development of the public distribution system, could be viewed as either coping mechanisms or as potential adaptations towards resilience, in that they seem to support the maintenance of the basic agroecological system even though it has lost productivity due to Lantana’s spread.

Other complementarities may also develop. For example, Lantana furniture production can potentially fill livelihood gaps in between quarrying and cropping, thus reducing vulnerability and maintaining forest livelihood resilience and values. Similarly, bamboo can be collected in opportunistic coordination with planned forest outings for livestock grazing (as with the Soliga) or to harvest Lantana for manufactures. Above all, these synergies between adaptation strategies require flexibility in response to dynamic social–ecological conditions and exigencies. Flexibility remains a central adaptive trait among smallholders in mixed economies and dynamic ecosystems (Netting 1993).

Coping with deleterious changes in biodiversity can affect human wellbeing in other important ways, including emotional states and cognitive orientations toward the future. Herders we spoke to in Kombudikki often seemed fatalistic about biodiversity change, predicting that, with Lantana’s continued spread, there would soon ‘be no more fodder’ for cattle in their traditional grazing areas. They would either have to migrate elsewhere with their herds, purchase fodder or pay others to graze animals, or give up forest grazing altogether. Giving up grazing entails converting most cattle to other assets, except perhaps for a few that can be kept close to home with minimal labour and feed—a common strategy among households that cannot afford to pay someone to graze cattle elsewhere. Relatively, few households can afford to purchase fodder or to send cattle to distant areas where fodder may still be abundant. Yet, giving up cattle altogether is hard to countenance, especially for Hindus who value cows as sacred possessions within a cultural system that treats them as sources of income, collateral, insurance, traction, fuel, fertilizer, milk, dowries, and other cultural purposes (Puri 2016). Thus, most families struggle to keep at least a few cattle to graze locally just to meet these needs.

Deteriorating forest conditions are sometimes understood as the result of supernatural malevolence toward humanity. The Lantana scourge may be explained as a kind of a curse that a ‘devil,’ or ‘evil god’ has visited upon the forest. Such perceptions, in conjunction with decreased forest access and visibility, exacerbate fears related to forest landscapes that were formerly associated with security, and the negative valence of Lantana as a cultural resource. Without a process of revitalization to adapt traditional values, knowledge, and practices towards new or newly proliferating resources amid changing biodiversity, human populations may become dependent on fewer assets and services within the ecosystem. In this respect, cognitive reorientation is especially important for adaptive behavioural change (cf. Wallace 1956). In the case of Lantana in the MM Hills, the loss of fodder and other highly valued NTFPs has catalysed increasing dependency on the state food distribution system, as well as increased market dependence (agricultural commodities and migratory wage labour) and, thus, entails greater vulnerability to their fluctuations. The public distribution subsidy means less dependence on agricultural success and less need for young men to return home from wage work to manage agricultural holdings, especially for Soliga. In this sense, the public distribution system could be viewed as indirectly subsidising the expansion of wage labour, an unanticipated contingency of intersecting top-down (public distribution system) and bottom-up (mobility and wage labour diversification) adaptation processes.

Thus, reductions in ecosystem services wrought by biodiversity change can pose multiple threats to human wellbeing. However, because the causal chains, scales of impact, and adaptation pathways are multiple and contingent, one cannot make simple predictions. For example, we must consider that limitations on human mobility and access to the forest are not merely a function of Lantana’s spread but also a result of political constraints such as increased restrictions and regulations around NTFP harvest imposed by the Forestry Service. In the past, restrictions were imposed by the reign of the outlaw brigand, Veerappan (Krupakar and Senani 2011), who is said to have exploited and controlled by violent means significant parts of the MM Hills and surrounding forest for his own gain. Such political forces may profoundly affect the development and adaptation of livelisystems, yet these can easily be overlooked if there is a myopic focus on balancing livelihood assets, functions, or community-based resource management institutions.

Lantana’s encroachment on forest vegetation and pastures will certainly have an effect on access to the natural resources that the local communities have depended on thus far. However, Lantana’s presence may create new opportunities for revitalising livelisystems. For example, Lantana’s toxic leaves are infused with strong chemicals that are repulsive and often toxic to herbivores (Sharma et al. 2007), making it a potentially useful hedge plant that can protect crops from free-ranging cattle and wild animals, so long as its spread can be controlled. Similarly, it has been argued that if Lantana is used in landscape management, it may be possible to enhance a range of ecosystem services at the landscape scale, for example, by keeping soil erosion under control while making use of Lantana as a hedge plant on hill slopes and field margins (Ganeshaiah and Shaanker 2001). The inclusion of Lantana in landscape management may also help to respond to ‘ecosystem disservices’ from, for example, crop-raiding animals. Lantana’s luxurious growth and production of woody stems can be exploited for charcoal-making, producing biogas and bio-ethanol, and making paper, baskets, and garden furniture (Sharma et al. 2005; Sugumaran and Seshadri 2009; Kuhad et al. 2010). This has already become part of local communities’ adaptation response, in some cases with support from NGOs. Furthermore, the chemicals contained in Lantana leaves have antibacterial, antimicrobial, anti-inflammatory and antitumor properties, meaning that the plant has considerable medicinal value (Sharma et al. 2007). This can provide opportunities for a local cottage industry in the manufacture of pharmaceutical products from Lantana. Such novel ecosystem products and services can help mitigate grazing and NTFP collection losses. However, assiduous human management may be required to find the optimal balance between Lantana and other forest resources, and to limit the plant’s potential for aggressive spread beyond its optimum boundaries.

## Rethinking adaptation in managing change in biocultural diversity

Given the above analysis, we suggest an approach to adaptation that helps to both integrate and expand the impact-risk, vulnerability, and pathway perspectives to more fully capture the dynamics of authocthonous adaptation to environmental change and its relation with human wellbeing. The *adaptation processes* approach suggests an alternative, beginning with an assessment of existing modes of adaptation and then focusing specifically on how these pathways have operated and evolved in relation to each other in the face of both environmental and social change, regardless of system or scale boundaries (Fig. 1).

The adaptation development cycle illustrated in Fig. 1 is designed as a set of queries to stimulate policy makers, researchers, and local actors alike to consider not only risk and vulnerability within specific geographic or economic sectors, but as well the full range of adaptation processes—past, present and future—that affect human-ecological and wellbeing conditions in a particular setting. A major advantage of the adaptation process framework is that it can avoid the problem of ‘projectised’ interventions, which have often ignored, distorted, or undermined local processes, agencies, and institutions, as development assistance and conservation interventions demonstrate (e.g. Honadle and Rosengard 1983; Nair 2014; Newsham and Bhagwat 2015; Macura et al. 2016; Bhagwat 2018). Similarly, attention to the relationship between local adaptation processes and both adaptation pathways and environmental change can help policy makers avoid reducing adaptation efforts to a few sociotechnical or developmental pathways (such as innovation or diversification) in response to risk or vulnerability, as is often the case. It does so by formalising the assessment of authocthonous adaptation in eight major processual forms, while at the same time recognising the diversity of adaptation trajectories that can develop within and between these pathways depending on local conditions.

The adaptation processes approach also encourages historical–ecological *backcasting* to capture a fuller understanding of how adaptation pathways have already evolved amid contingent social-ecological factors in specific landscapes over multiple time scales of (a) event; (b) cycle; and (c) the so-called *longue durée* (see Balée 2006). Indeed, the Lantana case necessitates examination of all three time scales in order to make informed judgements about future adaptation potential and co-evolution of social-ecological systems in the face of biodiversity and other change. Thus, if one considered only Lantana’s recent spread events, eradication might still seem to present a reasonable adaptation option, whereas a longer temporal perspective shows clearly that such a strategy is not practical (Bhagwat et al. 2012).

Similarly, the adaptation processes approach can be deployed in *forecasting* through scenario-building to consider how diverse processes and pathways might interact in response to future climate and biodiversity change. Just as it does not privilege a single process, the framework similarly does not privilege a single actor, such as the community, nation-state, or market, as the sole or primary locus of adaptation, but rather considers the range of actors from individual, household, community, regional, and national levels, and other agents within both the local ecosystem and the (typically supra-local) livelisystem.

At the same time, there are important caveats to using the adaptation pathway framework. First, particular pathways should not a priori be considered as adaptation processes. Rather, they are *responses* to environmental change that can lead to adaptations (see Puri 2015 for a discussion of the problems of establishing an action or events as a response). Second, the contingencies and complementarities of adaptation strategies in relation to pathways must be considered across a range of socio-environmental changes over time. Thus, reduced mobility in the forest could be considerably altered by an innovation such as a road, but the road itself might become a source of other stressors, adaptations and knock-on effects which might ultimately counter the benefits of increased forest mobility or access, for example by increasing forest competition. Third, the queries and responses posed in the adaptation cycle can be different for different actors or stakeholders at different scales. For example, in the MM Hills livelisystem, households with larger landholdings find it adaptive to deploy a segment of their labour force, specifically young men, into seasonal migratory wage labour (mobility and diversification) as strategy for reducing vulnerability and risks associated with poverty. Both cash and farm labour are needed for livelihoods but are now less compatible in the local environment since biodiversity changes have reduced wage opportunities from harvesting NTFPs and herding. In contrast, for households without such ties to the local agricultural base, mobility in the form of permanent migration to another village or urban centre may become the most attractive option. At still another level, from the perspective of a state supporting rural communities with subsidies, such as the public distribution system in Kombudikki, rural residents’ migration to cities with jobs and services might also be viewed as ‘adaptive,’ since it may ultimately be seen to reduce costs and dependency on state coffers (though migration also may stress urban environments; see the Foresight Report 2011). Consequently, adaptation studies must focus not only on social–environmental processes and pathways in the abstract, but also on the political ecology of actors and interests (Taylor 2014) at all relevant spatial scales, and how these may shape who is adapting or being expected to adapt, to what, how (adaptation pathways), and toward what ends.

A final advantage of the adaptation processes-to-pathways approach is that it is both general and particular. It is broad enough to permit comparison between human societies, yet place-specific in its ethnographic orientation towards the vast diversity of knowledge, values, and behaviour that defines human adaptive capacities, cultures, and careers around the world. This diversity is important, as every aspect of climate change is effectively mediated by cultural processes (Adger et al. 2013; Forsyth and Evans 2013). Correlatively, any viable culture must be able to respond adaptively to environmental change in situ and in vivo if it is to maintain a degree of distinctiveness. Biodiversity has been shown to be of critical importance to cultural diversity on earth (Maffi 2001, 2005; Maffi and Woodley 2010; Gorenflo et al. 2012), and diverse adaptations to changing biodiversity remain an imperative for maintaining both biocultural diversity and ecocultural health as sources of global and local resilience (Rapport and Maffi 2010), especially given the implications of recent planetary boundaries findings (Rockström et al. 2009; Steffen et al. 2015) in the emerging Anthropocene (Steffen et al. 2011).

In fact, with planetary boundaries already exceeded to the point of ‘high risk’ and the imperative to keep global average temperatures below 1.5 °C to avoid dangerous climate change, human adaptation can no longer be seen as separate from mitigation. Every significant mitigation effort has land use, biodiversity, and livelisystem adaptation implications. Thus, understanding synergies between mitigation and adaptation to environmental change is not only an imperative for sustaining human and planetary wellbeing, but also for the diversity of life that has allowed thousands of human societies to adapt and evolve in the first place. Lantana’s invasion is small and local by comparison. Yet, its widespread impacts on biodiversity and livelisystems make it a useful case for rethinking adaptation to environmental change.
